# Distinct Regions of the Large Extracellular Domain of Tetraspanin CD9 Are Involved in the Control of Human Multinucleated Giant Cell Formation

**DOI:** 10.1371/journal.pone.0116289

**Published:** 2014-12-31

**Authors:** Rachel S. Hulme, Adrian Higginbottom, John Palmer, Lynda J. Partridge, Peter N. Monk

**Affiliations:** 1 Department of Molecular Biology and Biotechnology, University of Sheffield, Sheffield, United Kingdom; 2 Department of Neuroscience, University of Sheffield Medical School, Sheffield, United Kingdom; 3 Department of Infection and Immunity, University of Sheffield Medical School, Sheffield, United Kingdom; University of Vermont, United States of America

## Abstract

Multinucleated giant cells, formed by the fusion of monocytes/macrophages, are features of chronic granulomatous inflammation associated with infections or the persistent presence of foreign material. The tetraspanins CD9 and CD81 regulate multinucleated giant cell formation: soluble recombinant proteins corresponding to the large extracellular domain (EC2) of human but not mouse CD9 can inhibit multinucleated giant cell formation, whereas human CD81 EC2 can antagonise this effect. Tetraspanin EC2 are all likely to have a conserved three helix sub-domain and a much less well-conserved or hypervariable sub-domain formed by short helices and interconnecting loops stabilised by two or more disulfide bridges. Using CD9/CD81 EC2 chimeras and point mutants we have mapped the specific regions of the CD9 EC2 involved in multinucleated giant cell formation. These were primarily located in two helices, one in each sub-domain. The cysteine residues involved in the formation of the disulfide bridges in CD9 EC2 were all essential for inhibitory activity but a conserved glycine residue in the tetraspanin-defining ‘CCG’ motif was not. A tyrosine residue in one of the active regions that is not conserved between human and mouse CD9 EC2, predicted to be solvent-exposed, was found to be only peripherally involved in this activity. We have defined two spatially-distinct sites on the CD9 EC2 that are required for inhibitory activity. Agents that target these sites could have therapeutic applications in diseases in which multinucleated giant cells play a pathogenic role.

## Introduction

The tetraspanins are a family of transmembrane glycoproteins, with thirty-three members identified in mammals [1]. Tetraspanins are characterised by four transmembrane domains, usually short intracellular N and C-termini, one small extracellular domain and one large extracellular domain (EC2) which has 2, 3 or 4 pairs of cysteine residues, with one pair in a highly conserved ‘CCG’ motif. The tetraspanins appear to have roles in many areas of cell biology, from cell motility, exosome formation and function, to cell fusion (reviewed in [2]–[4]) and can also form gateways for the invasion of cells by a wide range of pathogens (reviewed in [5], [6]). The tetraspanins are described as ‘molecular facilitators’ with the ability to influence the location and function of many membrane proteins including immunoglobulin superfamily proteins, proteoglycans, integrins, complement regulatory proteins, proteases, cadherins and G-protein coupled receptors [7]. Tetraspanins and partner proteins form tetraspanin enriched microdomains (TEM) [8] through a hierarchy of protein-protein interactions, with tetraspanins able to exist as homo- and heterodimers and also able to bind to the array of partner proteins. The existence of TEM have been inferred from experiments involving anti-tetraspanin antibodies [9], detergent extraction [10], recombinant tetraspanin fragments [11], Förster resonance energy transfer [12] and from single-molecule fluorescence microscopy [13], [14]. In addition, cryo-electron microscopy of two highly specialised tetraspanins, uroplakins 1a and 1b, which have an active role in the organisation of the urothelium [15], have helped define a possible structure for TEM [11], [13].

The EC2 domain has been shown to be critical for many of the interactions with partner proteins [16]–[18]. Crystal structures for the EC2 of one tetraspanin, CD81, show that it is organised into a ‘stalk’ with a globular ‘head’. The stalk and part of the head is formed by helices A, B, E in the CD81 EC2 structure, with an amino acid sequence that is relatively highly conserved between tetraspanin family members. This sub-domain is suggested to contain sites of tetraspanin-tetraspanin interaction whereas a second sub-domain (helices C, D in CD81 EC2), with greater heterogeneity in sequence and length between family members, may have more specific functional roles [19], [20]. It is this second ‘hypervariable’ region that contains the binding sites on tetraspanin CD81 for hepatitis C virus glycoprotein E2 [21] and B cell marker, CD19 [22]. The C-terminal half of the tetraspanin CD9 EC2, containing this hypervariable region, is also important for the interaction with the immunoglobulin superfamily member, EWI-2 [23]. Another interaction mapped to this sub-domain is that between mouse CD9 and pregnancy-specific glycoprotein PSG17 [24]. The same residues of CD9 (S173-F-Q) are also critical for the fusion of gametes during fertilisation, as are the cysteine residues involved in disulfide bridge formation [24]–[26].

The tetraspanins have been reported to be involved in a number of cell-fusion processes such as sperm∶egg fusion, muscle cell fusion, and virus-induced syncitial formation [27]–[29]. Of most relevance to the work detailed here are the recent reports of the role of tetraspanins in multinucleated giant cell (MGC) formation [30], [31]. MGC form as a result of macrophage fusion and are often referred to as ‘giant’ cells due to the large number of nuclei present in one cell. Multinucleation of macrophages provides them with enhanced destructive ability and due to their increased size allows them to break down larger components that could not be internalised by an individual cell [32]. MGC are commonly observed in granulomas characteristic of chronic inflammation where they usually have an average of ∼20 nuclei [33]. A particularly well documented pathology is that concerning the bacteria *Mycobacterium tuberculosis* [34]. The presence of MGC in granulomas has also been observed with infections such as leprosy and schistosomiasis [35], [36] and in inflammatory diseases such as sarcoidosis and giant cell arteritis [37], [38]. It has been reported that monoclonal antibodies to tetraspanins CD9 and CD81 but not CD63 enhance Con A-induced MGC formation from human monocyte precursors as well as human and murine alveolar macrophages. By contrast, a GST-CD9 EC2 fusion protein was found to inhibit MGC formation in a dose dependent manner [30]. Recent work in our laboratories [31] concurred with these findings except that we also identified a positive regulatory role for tetraspanin CD63, since a panel of anti-CD63 antibodies inhibited MGC formation. Recombinant EC2 proteins corresponding to CD9 and CD63 were also inhibitory whereas CD81 EC2 is not [31]. Interestingly, mouse CD9 EC2 had no effect on MGC formation by human monocytes, despite a high degree of sequence similarity [30], [31].

CD9 and CD81 EC2 are expected to have a similar structure because they are of a similar length, have the same number of cysteine residues [16] and both lack post-translational modification. Their different effects on MGC formation provided the opportunity to map the site or sites on CD9 EC2 involved in this process through the generation of a series of chimeric constructs. Constructs were assessed for gain of function (CD81 chimeras containing sections of CD9) or loss of function (CD9 chimeras containing sections of CD81). Two regions in different sub-domains of CD9 EC2 were shown to be important components of the inhibitory effect. Point mutations, designed on the basis of sequence differences between human and mouse CD9 EC2 or on known CD9 interactions sites, were used to further characterise these sites.

## Materials and Methods

### Production of GST-fusion proteins

Chimeric EC2 fusion proteins were produced by overlap extension PCR, with the swapped regions described in S1 Table in S1 File. All of the wild-type and point mutant EC2 fusion proteins, except Y148M and Y148A, were previously described [25]; the Y148 mutants were produced in the same way. The production and characterisation of the tetraspanin EC2 GST fusion proteins has been described in detail previously [25]. Briefly, tetraspanin EC2 regions that had been cloned into the pGEX-KG vector were expressed in Rosetta Gami B(DE3) *E. coli* (Merck Biosciences), culturing at 37°C for 4 hours after IPTG induction. Cells were pelleted and lysed by sonication in the presence of a protease inhibitor cocktail. Recombinant protein was purified in a single step by affinity chromatography on glutathione beads (Amersham-Pharmacia). Protein purity was analysed by Coomassie staining of SDS-PAGE gels and total protein concentration determined by Bradford assay. As it was not possible to separate the full-length EC2 fusion protein from the smaller fragments produced, the percentage of full length material in each sample was measured by densitometry. This was plotted against the inhibitory activity of each sample to ensure that inhibition of MGC formation was not a simple function of the concentration of the full length fusion protein (S1 Fig. in S1 File).

### Monocyte fusion assay

Peripheral blood monocytes were derived from peripheral whole blood of healthy volunteers by Ficoll-Hypaque density centrifugation as described elsewhere [24]. Briefly, mononuclear cells were seeded at 5×10^5^ cells/chamber in 0.5 ml RPMI-1640-10% FCS in an 8 chambered slide (Lab-Tek, Nunc). After overnight culture, adherent cells were cultured in RPMI containing 10% (v/v) foetal bovine serum in the presence or absence of 10 µg/ml Concanavalin A (Con A) (Sigma, UK) for 72 h at 37°C. The recombinant tetraspanin EC2 proteins were added at the stated concentrations at the same time as the Con A. In some cases 200 nM *E. coli* lipopolysaccharide (LPS; Sigma, UK) was used to determine if contaminants from the production process were responsible for effects observed. The cells were washed with PBS, fixed and permeabilised with acetone (5 min at room temperature), rehydrated with PBS then labelled with FITC-anti-CD63 (clone H5C6) and the nuclei counter-stained with propidium iodide (Sigma, UK). Fusion indices (number of nuclei within MGCs)/(total number of nuclei counted)×100) were determined by counting the number of nuclei in fused cells (>5 nuclei per cell) and unfused cells in 6 randomly chosen fields (2 chambers per donor, minimum of three donors per experiment) using a Nikon Eclipse E400 immunofluorescence microscope. The numbers of nuclei per MGC were recorded and the average nuclei per MGC calculated. Counts from each chamber are presented as separate data points.

### Ethics statement

The study was approved by the South Sheffield Research Ethics Committee (protocol number SSREC/02/299). Participants provided written consent and records have been retained by the named researchers on the Ethics Protocol, as required by the Research Ethics Committee.

## Results

### Design and expression of chimeric and mutant EC2 proteins

**Figure 1:**
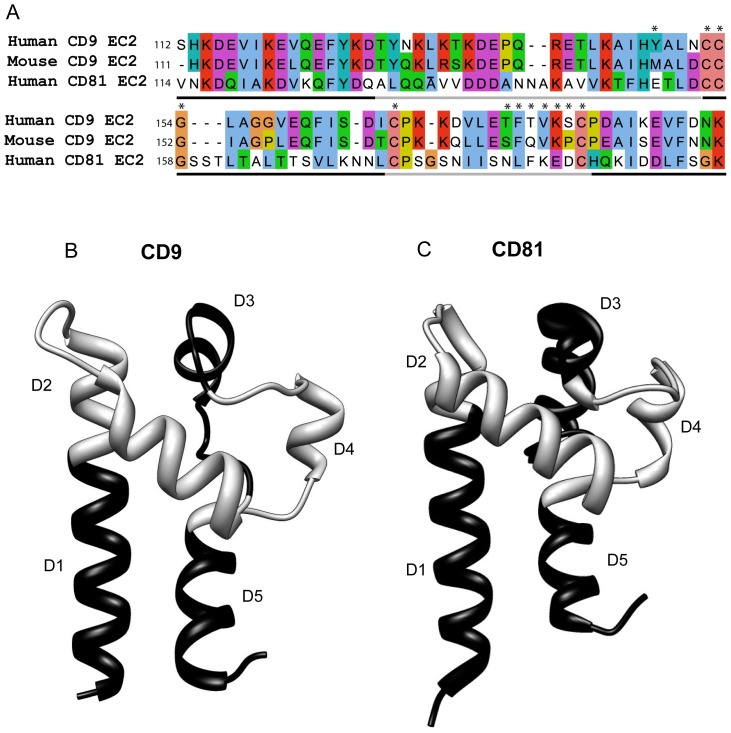
Comparison of CD9 and CD81 sequences and structures. Fig. 1A: sequences for the large extracellular domains (EC2) of human CD9 and CD81 and mouse CD9, aligned using ClustalW [49] in JalView [50]. Conserved residues are coloured according to physicochemical properties (http://www.ebi.ac.uk/Tools/msa/clustalw2/help/faq.html#24). Asterisks show residues that were mutated and the gray/black line indicates regions that were exchanged to form chimeric EC2 fusion proteins. Fig. 1B, C: Structures of CD9 (modelled on CD81 EC2 (PDB IV5) using I-TASSER [51]) and CD81 (from PDB file 1V5 [18]) and, showing regions exchanged in the production of the chimeras in alternating black and gray, as in Fig. 1A. Structures visualised using the UCSF Chimera package, developed by the Resource for Biocomputing, Visualization, and Informatics at the University of California, San Francisco, funded by grants from the National Institutes of Health National Center for Research Resources (2P41RR001081) and National Institute of General Medical Sciences (9P41GM103311) [52].

The sequences of human CD9 and CD81 EC2 are shown in Fig. 1A, along with the regions that were exchanged between the two proteins (light and dark gray lines). The crystal structure of CD81 EC2 [16] and a putative structure for CD9 (modelled on CD81 EC2) are shown in Fig. 1B. Chimeras were designed to exchange most of the two helical stalk helices (chimeras D1, D5) and the three helices in the head subdomain (D2, D3, D4). Finally, chimera D6 exchanged both of the smaller helices (D3, D4) simultaneously (Fig. 1B). The exact sites of the exchanges are shown in S1 Table in S1 File. GST fusion proteins with the EC2 constructs were expressed and affinity purified as described. SDS-PAGE analysis shows the proportion of each preparation that was at the expected apparent molecular weight (S1 Fig. in S1 File). Point mutants (apart from Y148A, Y148M and G154A) have been previously reported [25].

### Effect of tetraspanin EC2 proteins on MGC formation

**Figure 2:**
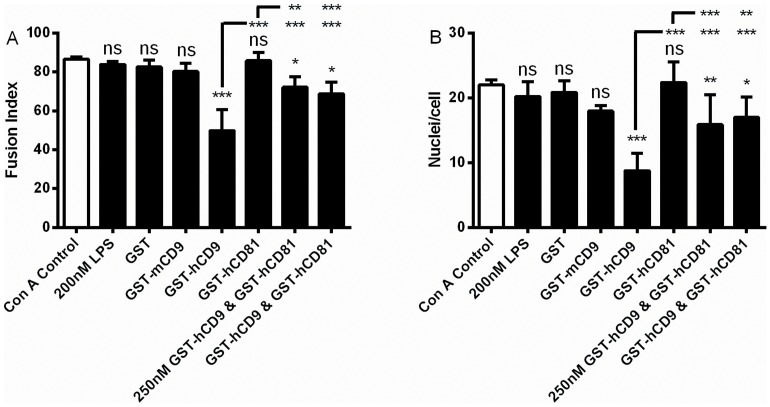
Effects of 500 nM EC2 domains on multinucleate giant cell formation. Fig. 2 A, B shows the effects on fusion index and average number of nuclei per giant cell (>5 nuclei per cell), respectively. Monocytes were treated with Con A alone, or with Con A and 200 nM lipopolysaccharide, 500 nM GST or the indicated recombinant tetraspanin EC2 GST fusion protein, except for the data indicated where monocytes were treated with Con A and 250 nM each of the respective EC2 protein. Data are the means of at least 6 experiments ± SEM. Significance was calculated using one way ANOVA with Bonferroni post-test; p values ***:<0.0001; **: <0.01; *: <0.05. Unless otherwise indicated, the significance of the difference from the Con A control (clear bar) is shown.

In the presence of Con A, monocytes fuse to become MGC and the effects of GST-tetraspanin CD9 and CD81 EC2 domains on this phenomenon have previously been published [30], [31]; data are shown here for the purposes of comparison. Fusion indices were 81–89% and the number of nuclei present in a giant cell ranged from 2 to 27 with a mean of 22 (Figs. 2A, B). Relative to GST alone, human CD9 EC2 but not mouse CD9 or human CD81 EC2 could inhibit fusion by ∼40% (Figs. 2A, B). The bacterial endotoxin, lipopolysaccharide, (LPS), present because an *E. coli* system was used to express the GST-fusion proteins, was tested for any effect on MGC formation. No effect was observed on fusion index or the average number of nuclei within a giant cell. Co-incubation of CD9 and CD81 EC2 proteins, at either 250 nM or 500 nM of each protein, caused significantly less inhibition relative to CD9 EC2 alone (Figs. 2A, B), suggesting that CD81 EC2 is not inactive but can actually antagonise the effect of CD9 EC2 on monocyte fusion.

### Effect of chimeric tetraspanin EC2 proteins on MGC formation

**Figure 3:**
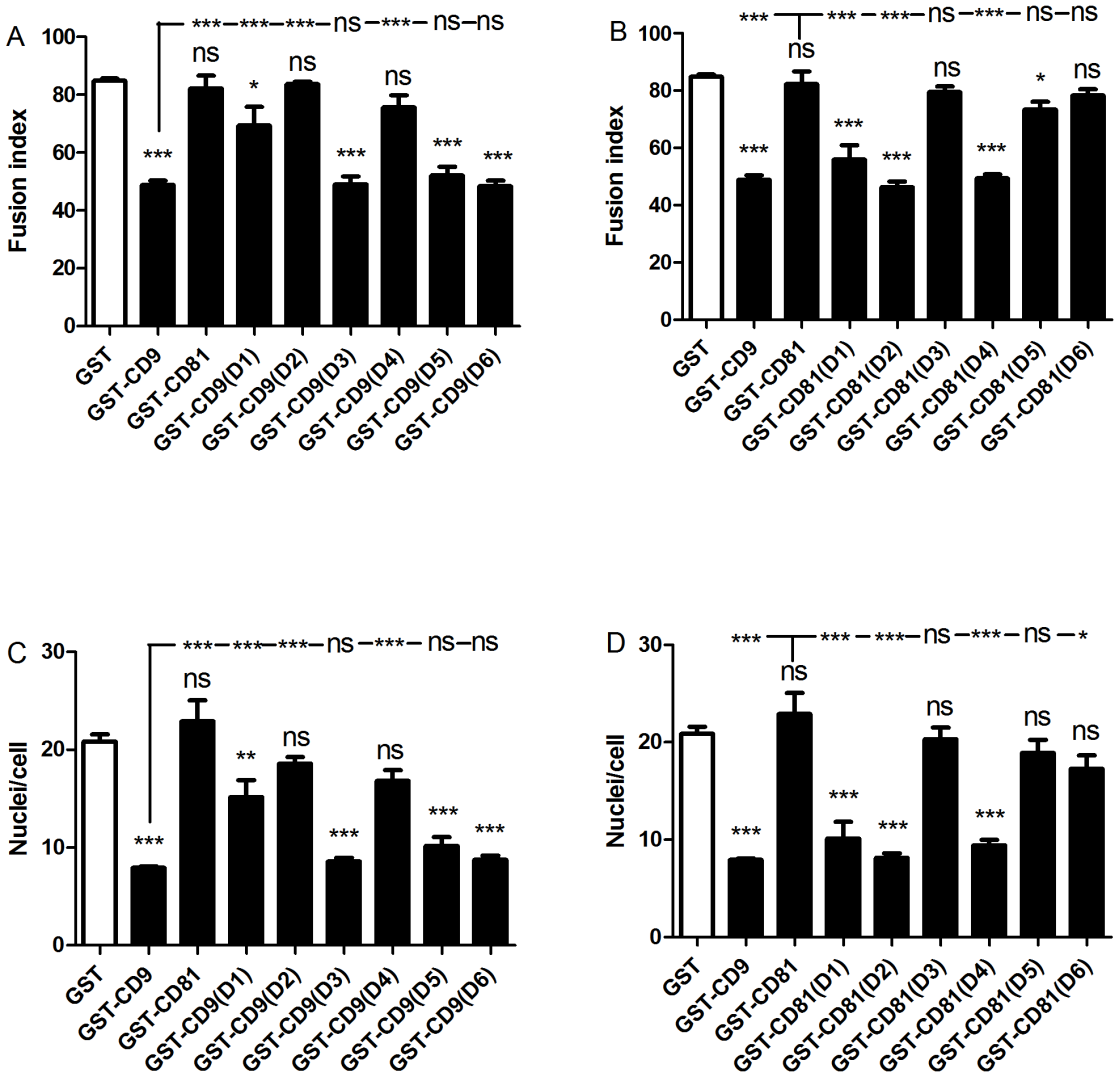
Effects of 500 nM CD9/CD81 chimeric EC2 domains on multinucleate giant cell formation. Fig. 3 A, B shows the effects on fusion index and average number of nuclei per giant cell (>5 nuclei per cell), respectively. Monocytes were treated with Con A and 500 nM GST or 500 nM of the indicated recombinant chimeric EC2 GST fusion protein, in which different CD81 sequences were used to replace the relevant CD9 sequence. Fig. 3 C, D shows the effects on fusion index and average number of nuclei per giant cell (>5 nuclei per cell), respectively. Monocytes were treated with Con A and 500 nM GST or 500 nM of the indicated recombinant chimeric EC2 GST fusion protein, in which different CD9 sequences were used to replace the relevant CD81 sequence. Data are the means of 6 experiments ± SEM. Significance was calculated using one way ANOVA with Bonferroni post-test; p values ***:<0.0001; **: <0.01; *: <0.05. Unless otherwise indicated, the significance of the difference from the GST only control (clear bar) is shown.

**Figure 4:**
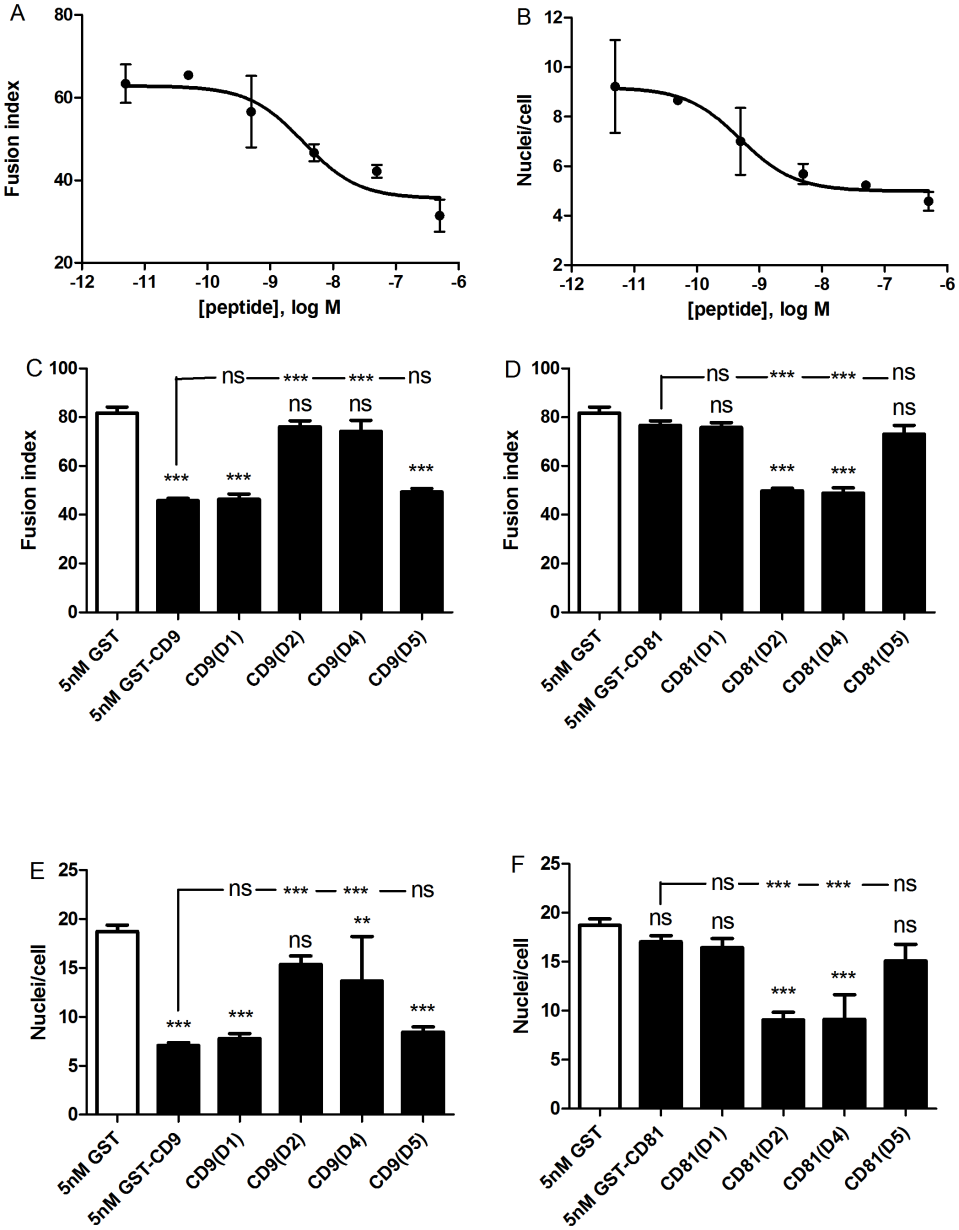
Effects of 5 nM CD9/CD81 chimeric EC2 domains on multinucleate giant cell formation. Fig. 4 A, B shows the effects on fusion index and average number of nuclei per giant cell (>5 nuclei per cell), respectively, of increasing concentrations of human CD9 EC2 GST fusion protein. Data are the means of 2 experiments ± SEM. Fig. 4 C, D shows the effects on fusion index and average number of nuclei per giant cell (>5 nuclei per cell), respectively. Monocytes were treated with Con A and 5 nM GST or 5 nM of the indicated recombinant chimeric EC2 GST fusion protein, in which different CD81 sequences were used to replace the relevant CD9 sequence. Fig. 4 E, F shows the effects on fusion index and average number of nuclei per giant cell (>5 nuclei per cell), respectively. Monocytes were treated with Con A and 5 nM GST or 5 nM of the indicated recombinant chimeric EC2 GST fusion protein, in which different CD9 sequences were used to replace the relevant CD81 sequence. Data in Fig. 4C–F are the means of 6 experiments ± SEM. Significance was calculated using one way ANOVA with Bonferroni post-test; p values ***:<0.0001; **: <0.01; *: <0.05. Unless otherwise indicated, the significance of the difference from the GST only control (clear bar) is shown.

The twelve chimeric constructs of CD9/CD81 EC2 domains were assessed alongside controls (GST alone, wild type CD9 and CD81 EC2 proteins) at 500 nM, a dose of human CD9 EC2 previously shown to inhibit MGC formation [31]. Chimeras were assessed for a loss of inhibitory effect when inserting CD81 sites into the CD9 EC2 and a gain of inhibitory effect when CD9 sites were inserted into CD81 EC2. Figs. 3A–D illustrate the effects of the chimeric constructs on fusion index and giant cell size. Two sites on CD9 EC2 appeared to be essential to fusion: when D2 or D4 were replaced by the corresponding region of CD81 EC2, the inhibitory effect of CD9 EC2 was lost (Figs. 3A, C). In the reciprocal chimeras, these regions also significantly enhanced biological activity when inserted into the CD81 EC2 (Figs. 3B, D). The substitution of CD81 D1 into CD9 EC2 had a small negative effect on activity and was significantly different to wild type CD9 EC2; conversely, the CD81 EC2 chimera containing CD9 D1 significantly inhibited fusion and MGC size (Figs. 3A–D). The CD81 chimera containing CD9 D5 slightly inhibited the fusion index but this was not significant relative to wild type CD81 EC2 and there was no effect on MGC size. The corresponding CD9 chimera (containing CD81 D5) was as active as wild type CD9 EC2. Unexpectedly, the CD9 chimera containing the CD81 D6 region inhibited fusion whereas the reverse CD81chimera (containing CD9 D6) was inactive, despite CD81 D6 containing the CD9 D4 loop that was shown to confer activity when present in CD81. To help determine if ‘stalk’ regions D1 and D5 of CD9 EC2, which showed weak inhibitory activity in CD81 chimeras, are required for the inhibition of MGC formation, the assays were repeated at a lower concentration of recombinant protein. A dose-response curve indicated that 5 nM CD9 EC2 would provide a sub-maximal effect (Fig. 4A, B). At 5 nM, substituting either D2 or D4 of CD81 into CD9 EC2 completely eliminated inhibitory activity whereas D1 and D5 had no effect (Figs. 4C–F). In the reciprocal chimeras, D2 and D4 caused a gain-of-function in CD81 EC2, whereas D1 and D5 had no effect. From these experiments, we can conclude that D2 (the top of the N-terminal stalk and the first helix of the head sub-domains) and D4 (the third of the head helices) are critical for the inhibitory activity of CD9 EC2 on MGC formation. D1 (the major part of the N-terminal stalk helix) may have a minor role, whereas D3 (the second head helix) and D5 (the C-terminal stalk helix) are not involved.

### The effects of point mutations in D2 and D4 on the inhibitory activity of CD9 EC2

**Figure 5:**
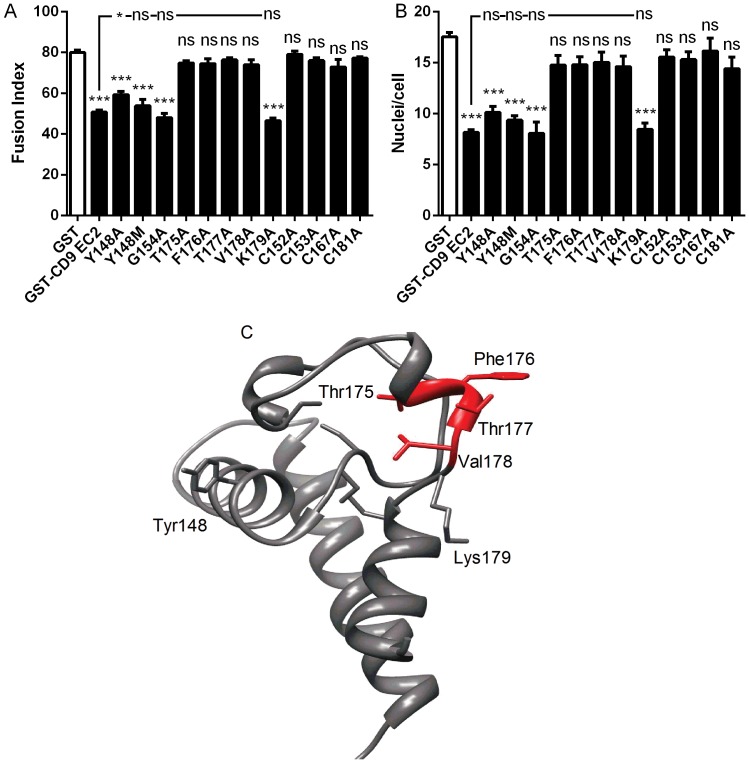
Effects of point mutations on the ability of CD9 EC2 to inhibit multinucleate giant cell formation. Fig. 5 A, B shows the effects on fusion index and average number of nuclei per giant cell (>5 nuclei per cell), respectively. Monocytes were treated with Con A and 500 nM GST or 500 nM of the indicated recombinant mutant CD9 EC2 GST fusion protein. Data are the means of at least 6 experiments ± SEM. Significance was calculated using one way ANOVA with Bonferroni post-test; p values ***:<0.0001; **: <0.01; *: <0.05. Unless otherwise indicated, the significance of the difference from the GST only control (clear bar) is shown. Fig. 5C shows the positions of selected mutations on the model of CD9 EC2 protein, with the important T175-V178 region highlighted in red.

As mouse CD9 EC2 is inactive in the MGC formation assay, the sequences of mouse CD9 and human CD9 EC2 D2 and D4 regions were compared (Fig. 1A). In the D2 site of human CD9, five residues were different in mouse CD9, with only one substantial side-chain difference at Y148, which is M in mouse CD9 EC2. This residue, corresponding to E150 in human CD81, is predicted to be solvent exposed in the model of CD9 EC2 (Fig. 5C) and so was selected for mutation. In D2, the mutant Y148A had only a small effect on the Fusion Index relative to wild type human CD9 EC2 and none on giant cell size while Y148M was identical to wild type (Fig. 5A, B), suggesting that this reside is not directly involved in the inhibitory activity. In the D4 site of human CD9, five residue differences were identified although none showed major changes in charge or size. However, residues in this region have previously been shown to be important in sperm/egg fusion [25], [26] and so point mutants were tested (T175A to K179A). The effects of the point mutants on MGC fusion rates and size were determined at 500 nM (Figs. 5A, B). The K179A mutation did not affect human CD9 EC2 inhibition of MGC formation or size whereas other mutations within the D4 exchange regions completely abrogated inhibition.

### Effects of conserved C and G residues in human CD9 EC2

Other point mutants tested in the fusion assay included the four cysteine residues and the G154 residue in the ‘CCG’ motif, characteristic of the mammalian tetraspanins (Fig. 5A, B). The mutation of any of the four C residues (C152, C153, C167, C181) led to the complete loss of inhibitory activity, even at 500 nM, indicating that correct formation of the disulfide bridges are absolutely required for the human CD9 EC2 to inhibit MGC formation. The ‘CCG’ motif is conserved in nearly all tetraspanins but mutation of G174 to A had no effect on the inhibitory activity of CD9 EC2, indicating that a residue with a small side-chain is tolerated at this position.

## Discussion

Consistent with previous findings, GST-CD9 EC2 significantly inhibited size and fusion rates of MGC (data presented here and [30], [31]). Although we have not attempted to investigate the mechanism of this activity here, it may be similar to the effects of tetraspanin EC2 proteins on the attachment of leukocytes to endothelial cells. EC2 proteins inhibit this activity by decreasing the clustering of adhesion molecules, such as VCAM-1 and ICAM-1 [11]. Neither human CD81 EC2 nor mouse CD9 EC2 inhibited MGC formation, indicating that sequence specificity is important for this activity. Human CD9 EC2 shares 77% sequence identity with mouse CD9 EC2 and ∼23% identity with human CD81 EC2. The absence of inhibitory activity of CD81 EC2 cannot be attributed to incorrect folding as this has been assessed using conformation-sensitive antibodies in Western blotting and immunoprecipitation [31]. Functional activity of the GST-CD81 EC2 produced in this laboratory has also been established in other biological systems, where it has been shown to inhibit HCV binding to hepatocytes [21] and macrophage infection by HIV-1 [39]. Interestingly, CD81 EC2 was also not simply inactive but was capable of antagonising the activity of CD9 EC2. The molecular basis for this is unknown but suggests the interaction of different tetraspanins with positive and negative regulators of fusion. This view is supported by the activities of anti-tetraspanin antibodies on MGC formation: anti-CD63 antibodies can block fusion whereas anti-CD9 and anti-CD81 antibodies promote fusion [30], [31]. The lack of activity of region D6 (which in CD9 EC2 contains an ‘active’ region capable of inhibiting fusion) in either of the exchanges might also suggest that the control of MGC formation by tetraspanins is not a fixed property. This implies that the control of fusion by tetraspanins might be switchable by changes of conformation in the EC2 region, as previously observed in the tetraspanin CD63 control of mast cell degranulation. Thus the hypervariable D3 and D4 regions of CD81 may have the potential to inhibit fusion in certain conformations, for example when constrained by the scaffold of CD9.

**Figure 6:**
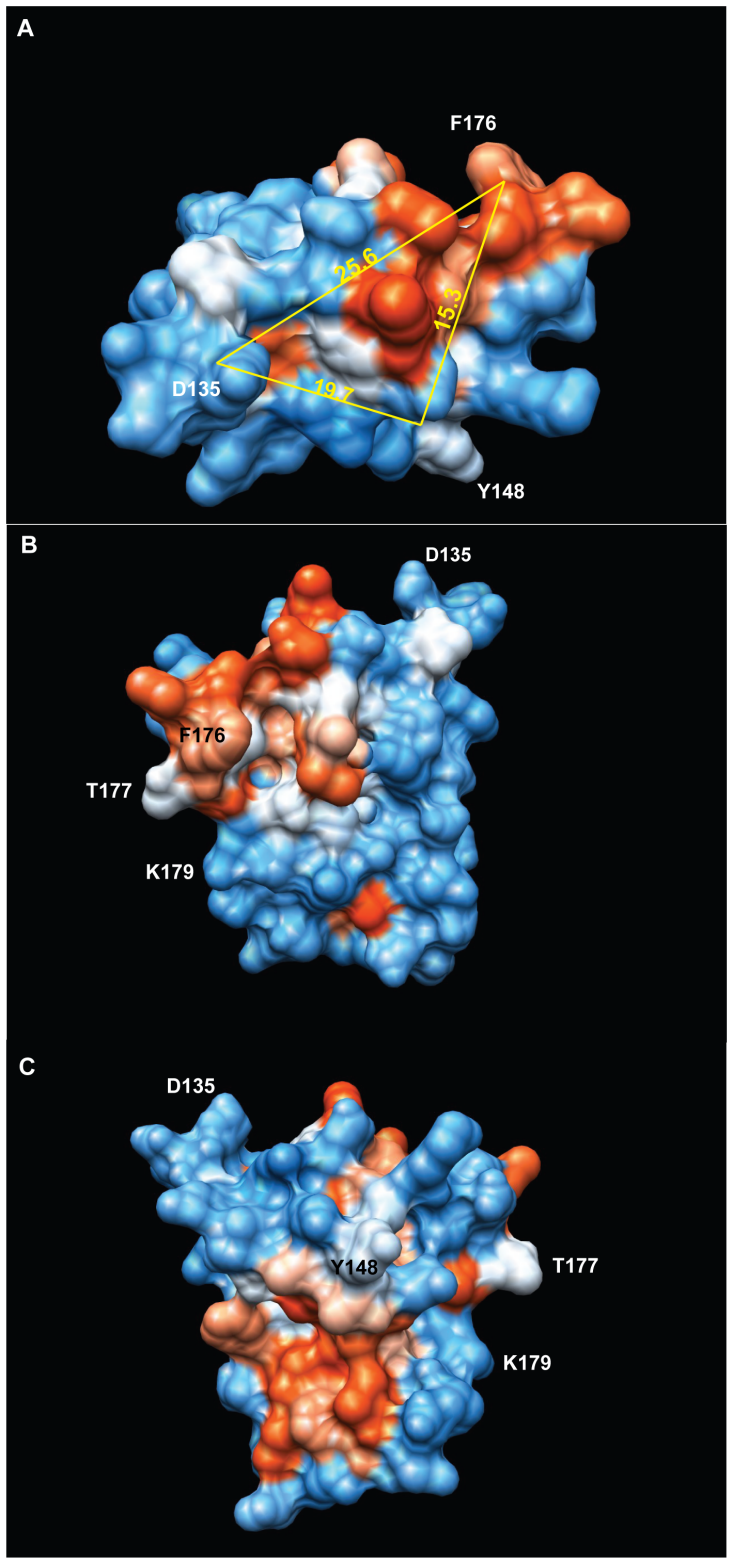
Depiction of the regions of CD9 EC2 involved in the inhibition of multinucleate giant cell formation. Structure of CD9 (modelled on CD81 EC2 (PDB IV5) using I-TASSER [51]), with surface polarity depicted from low (red) to high (blue). Distances between residues involved in the inhibitory effect (F186, Y148) or at the N-terminal end of the scond (B) helix (D135) are shown in Å, measured from the backbone amide N atom. Structure visualised using the UCSF Chimera package, developed by the Resource for Biocomputing, Visualization, and Informatics at the University of California, San Francisco, funded by grants from the National Institutes of Health National Center for Research Resources (2P41RR001081) and National Institute of General Medical Sciences (9P41GM103311) [52].

Using CD9/CD81 EC2 chimeras, we have identified two distinct regions of CD9 EC2 that are essential for inhibition of MGC formation (Fig. 6). These regions encompass the relatively well-conserved B helix preceding the CCG motif and the loop that connects it to the ‘stalk’ helix A (chimera D2) and the first sub-loop containing helix C within the ‘hypervariable’ region (chimera D4) [20]. The critical residues in these regions have not been systematically investigated and so we do not know if these regions form a single extended interaction site or two (or more) separate sites. Y148 and D135 at the C and N-terminal ends of helix B are ∼15 Å and ∼26 Å away from the amine N atom of F176, a residue that is required for activity (Fig. 6). The potential binding surfaces defined by these residues are composed of a hydrophobic ‘patch’ and a more polar region along helix B. The conserved head domain of CD81 EC2 contains a single region critical for *Plasmodium* infection of hepatocytes, mapped to the acidic residues in the loop that joins the helices A and B (at the same position as D135 of CD9 EC2, in the D2 exchange region) and a number of residues (T149, F150, T153, L154) aligned on the outer face of CD81 EC2 helix B (flanking the position of Y148 in CD9 EC2) [41]. A tetraspanin from *Schistosoma japonicum*, sjc23, can bind human IgG at a single site immediately preceding the CCG motif and synthetic peptides carrying the sequence KIQTSFHCC (most of helix B) were found to block binding [42]. In the hypervariable region, there are also several examples of binding sites. The mutation of T175, F176 or V178 in within the second sub-loop of human CD9 EC2 (analogous to S173-Q175 in mouse CD9) prevents the inhibition of sperm/oocyte fusion by GST-CD9 EC2 [25], [26]. L173-K192 of human CD9 EC2 has also been shown to form a binding site for fibronectin [43]. F186 in the same region of human CD81 EC2 is essential for binding of the envelope glycoprotein E2 in Hepatitis C virus, perhaps forming part of a hydrophobic patch involving I181, I182 and L185 [21]. CD151 potentially has an extra disulfide bridge in the EC2 that could provide a more complex sub-loop structure. Residues 186–217, including the sequence QRD, form a binding site for α3β1 integrin, promoting an interaction that is resistant to most detergents [44], [45]. Unlike the other activities so far defined for tetraspanins, the inhibition of MGC formation requires a widely distributed site (or sites) on CD9 EC2, suggesting that the soluble EC2 interacts with two or more proteins, perhaps acting to remove them from TEM or to hold them in an unfavourable orientation. Native CD9, anchored in a TEM, may interact with the same proteins, thus functioning as a negative regulator of fusion, as reported in several studies [29], [30], [46], [47]. In contrast, CD9 has a permissive role in sperm∶egg fusion (reviewed in [48]), suggesting differences in the fusion mechanisms used by different cell types.

Mutation of a number of residues in the D2 and D4 sites of CD9 EC2 resulted in the loss of inhibitory activity to varying extents. Residues in the D4 region of CD9 have previously been implicated in sperm∶egg fusion [25], [26]. Mutations here (with the exception of K179A) result in loss of inhibition in the present study, suggesting that the CD9 EC2 site required for sperm/egg fusion is also involved in MGC formation. It is interesting to note that K179 of recombinant CD9 EC2 is not involved in sperm/egg fusion but is required for the inhibition of the adhesion of the sperm, although the molecules that CD9 interacts with on the egg cell surface are still unknown. Mutation of the cysteine residues of CD9 EC2 demonstrated that intact disulfide linkages are required for the inhibitory effect on MGC formation, indicating that correct folding is necessary. We have also observed this for sperm/egg fusion and for recognition of EC2 by conformation-sensitive antibodies [21], [25]. The lack of an inhibitory effect of murine CD9 EC2 is not explicable in terms of the substitution of methionine at Y148 in the mouse protein, although alanine was not as well tolerated. This suggests that the overall structure of the D2 region, which contains two helices and a loop, might be important. Similarly, the inability of CD9 D6 (which contains both D3 and D4 loops) to transfer of inhibitory activity to CD81 whereas D4 alone is strongly active also suggests a significant structural contribution of D3 to the suppression of D4 activity.

## Conclusions

The functional sites on human CD9 EC2 that are required for the inhibition of MGC formation have been mapped to two separate regions, both which are required for activity. Compounds that interfere with the activity of these sites may be useful therapeutic agents that can block the formation of MGC in pathological conditions such as giant cell arteritis.

## Supporting Information

S1 File Contains the following files: **S1 Fig.** The relation between percentage of full length fusion protein and ability to inhibit MGC formation. Fig S1A is a graphical representation of the level of protein present in the major 35–36 kDa tetraspanin band on SDS-PAGE plotted against the percentage inhibition of MGC fusion at 500 nM total protein concentration. Fig. S1B is a representative SDS-PAGE experiment, showing the full-length GST fusion protein indicated by the arrow on the right and with the percentage of each chimera at full length, measured by densitometry, shown in each lane. **S1 Table.** Details of chimeras.
(DOCX)
