# Supplementary material for: Distinct Regions of the Large Extracellular Domain of Tetraspanin CD9 Are Involved in the Control of Human Multinucleated Giant Cell Formation
Source: PLoS One. 2014 Dec 31;9(12):e116289. doi: 10.1371/journal.pone.0116289 (PMC4281222; doi:10.1371/journal.pone.0116289)
Supplement: S1 File — Contains the following files: S1 Fig. The relation between percentage of full length fusion protein and ability to inhibit MGC formation. Fig S1A is a graphical representation of the level of protein present in the major 35–36 kDa tetraspanin band on SDS-PAGE plotted against the percentage inhibition of MGC fusion at 500 nM total protein concentration. Fig. S1B is a representative SDS-PAGE experiment, showing the full-length GST fusion protein indicated by the arrow on the right and with the percentage of each chimera at full length, measured by densitometry, shown in each lane. S1 Table. Details of chimeras. (DOCX) [file pone.0116289.s001.docx]

**Design of chimeras**

Table S1 shows the positions of the sequences exchanged to form the chimera. CD81 EC2 is 8 residues longer than CD9 EC2, including an extra turn on each of helices A and B and 3 extra residues in the sub-loop following the CCG motif. Chimeras with site D1 exchanged (most of helix A) were of unequal length, to accommodate part of this size disparity and to retain all of the residues thought to be involved in inter-chain bonding networks in this helix [16]. The D2 exchange sites were of equal length as this region is tethered at both ends by helix A and the disulfide linkages of the CCG motif. D3, D4 and D6 exchange sites were defined naturally by the position of the cysteine residues and D5 was imply the remainder of the EC2 domains after the final conserved cysteine, which forms all of helix E.

**Quality of EC2 proteins**

The quality of the EC2 proteins was assessed by SDS-PAGE and western blotting. Purified fractions of GST-CD9 EC2 and GST-CD81 EC2 on SDS-PAGE (Figure 1B) contained some truncated protein and free GST (at ~25kDa) and the fused GST-tetraspanin EC2 proteins apparent between 35 - 36kDa. A number of bands were visible for the GST-EC2 proteins of CD9 and CD81 where the upper band is the full length protein, as determined by western blotting with specific anti-CD9 or CD81 antibodies (not shown). Lower molecular weight material could be attributed to endogenous protease activity (despite the use of protease inhibitors during purification) or free GST at ~25kDa. Densitometric analysis of SDS-PAGE was performed to measure the percentage of protein found at the correct length for all chimeras and mutants except for the Cys mutants, in which the correct molecular weight bands could not be identified by western blot [25]. The degree of inhibition of MGC formation is plotted against the percentage of protein in this band for each preparation (Fig S1). There is no correlation between these two variables, confirming inhibition is not a simple effect of protein concentration but depends critically on certain sequences in CD9 EC2. LPS contamination ranged from 0-53ng/ml at the dilution at which the EC2 proteins were used, well below the concentration likely to affect MGC formation and was present in both inhibitory and non-inhibitory proteins.

**Table S1. Details of chimeras.**

| **Chimera** | **Residue numbering of insert** | **Number of amino acids in insert (total number)** |
| --- | --- | --- |
| Native CD81 EC2 | Full length: F113-K201 | (89) |
| CD81 D1 | CD9 S112-D127 | 16 (86) |
| CD81 D2 | CD9 T128-N153 | 24 (89) |
| CD81 D3 | CD9 C152-I166 | 15 (85) |
| CD81 D4 | CD9 C168-C181 | 15 (88) |
| CD81 D5 | CD9 P182-K192 | 11 (89) |
| CD81 D6 | CD9C152-C181 | 30 (84) |
| Native CD9 EC2 | Full length: S112-K192 | (81) |
| CD9 D1 | F113-K131 | 19 (84) |
| CD9 D2 | Q132-D155 | 24 (81) |
| CD9 D3 | C156-K174 | 19 (85) |
| CD9 D4 | C175-C190 | 16 (82) |
| CD9 D5 | H191-K201 | 11 (81) |
| CD9 D6 | C156-C190 | 35 (86) |

**
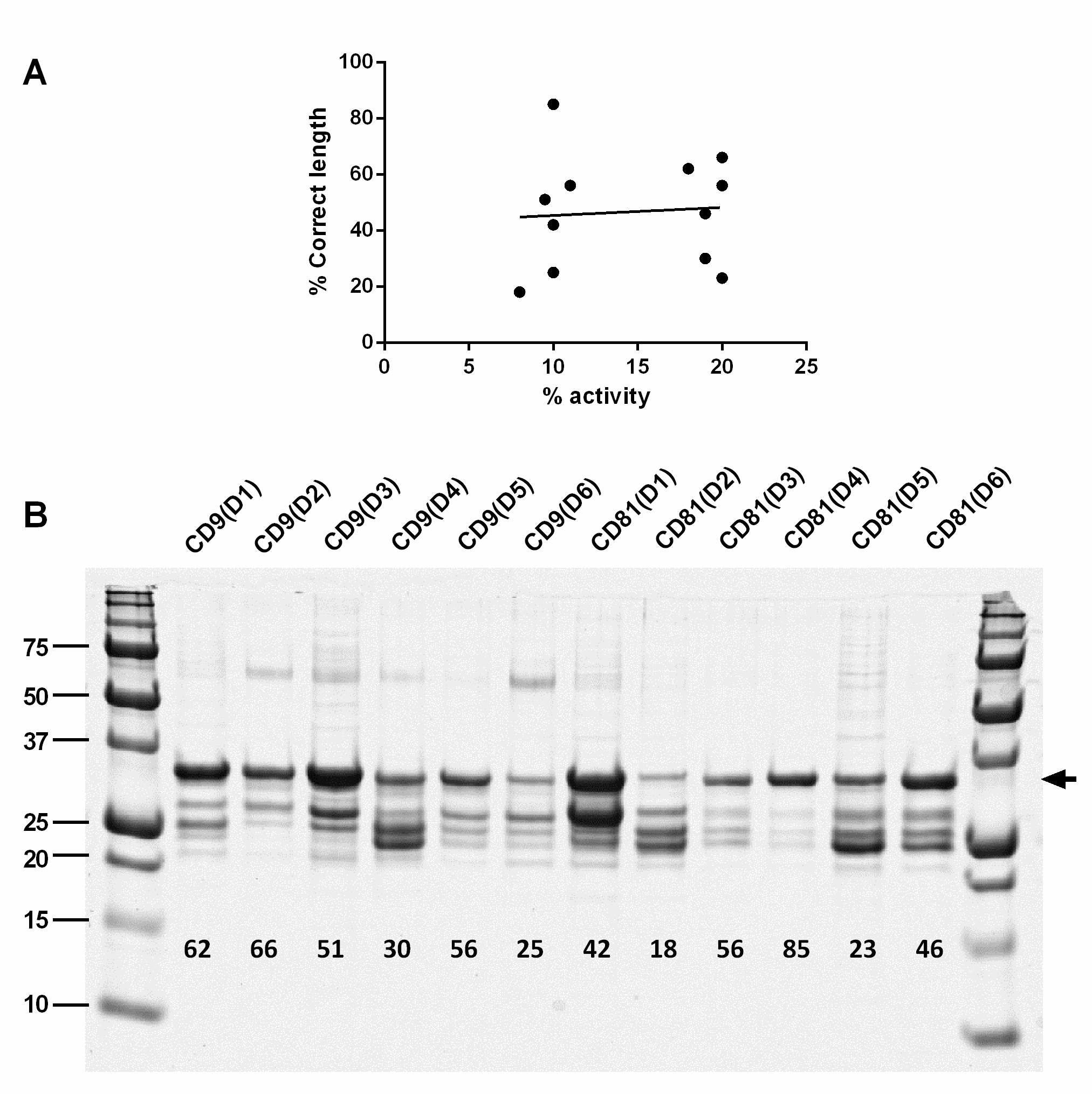
**

**Figure S1. The relation between percentage of full length fusion protein and ability to inhibit MGC formation.** Fig S1A is a graphical representation of the level of protein present in the major 35-36kDa tetraspanin band on SDS-PAGE plotted against the percentage inhibition of MGC fusion at 500nM total protein concentration. Fig S1B is a representative SDS-PAGE experiment, showing the full-length GST fusion protein indicated by the arrow on the right and with the percentage of each chimera at full length, measured by densitometry, shown in each lane.
